# Baroreflex Sensitivity as a Surrogate Biomarker for Concurrently Assessing the Severity of Arterial Stiffness and Cardiovascular Autonomic Neuropathy in Individuals with Type 2 Diabetes

**DOI:** 10.3390/jpm14050491

**Published:** 2024-05-03

**Authors:** Dong-Yi Hsieh, Yun-Ru Lai, Chih-Cheng Huang, Yung-Nien Chen, Szu-Ying Wu, Wen-Chan Chiu, Ben-Chung Cheng, Ting-Yin Lin, Hui-Ching Chiang, Cheng-Hsien Lu

**Affiliations:** 1 Department of Neurology, Kaohsiung Chang Gung Memorial Hospital, Chang Gung University College of Medicine, Kaohsiung City 83301, Taiwan; b9202095@cgmh.org.tw (D.-Y.H.); yunrulai@cgmh.org.tw (Y.-R.L.); may690210@cgmh.org.tw (H.-C.C.); 2 Department of Hyperbaric Oxygen Therapy Center, Kaohsiung Chang Gung Memorial Hospital, Chang Gung University College of Medicine, Kaohsiung City 83301, Taiwan; 3 Department of Neurology, Chi-Mei Medical Center, Tainan City 73657, Taiwan; hjc2828@gmail.com; 4 Department of Internal Medicine, Kaohsiung Chang Gung Memorial Hospital, Chang Gung University College of Medicine, Kaohsiung City 83301, Taiwan; b9502055@cgmh.org.tw (Y.-N.C.); qwerty@cgmh.org.tw (W.-C.C.); benz@cgmh.org.tw (B.-C.C.); 5 Department of Chinese Medicine, Kaohsiung Chang Gung Memorial Hospital, Chang Gung University College of Medicine, Kaohsiung City 83301, Taiwan; rickywu@cgmh.org.tw; 6 Department of Nursing, Kaohsiung Chang Gung Memorial Hospital, Chang Gung University College of Medicine, Kaohsiung City 83301, Taiwan; s971078@cgmh.org.tw; 7 Department of Center for Shockwave Medicine and Tissue Engineering, Kaohsiung Chang Gung Memorial Hospital, Chang Gung University College of Medicine, Kaohsiung City 83301, Taiwan; 8 Department of Biological Science, National Sun Yat-Sen University, Kaohsiung City 80424, Taiwan; 9 Department of Neurology, Xiamen Chang Gung Memorial Hospital, Xiamen 361126, China

**Keywords:** arterial stiffness, baroreflex sensitivity, brachial–ankle pulse wave velocity, cardiovascular autonomic neuropathy, composite autonomic scoring scale, type 2 diabetes mellitus

## Abstract

This study aimed to investigate whether baroreflex sensitivity (BRS) could serve as a reliable metric for assessing cardiovascular autonomic neuropathy (CAN) and concurrently act as a surrogate biomarker for evaluating the severity of arterial stiffness and CAN in individuals diagnosed with type 2 diabetes mellitus (T2DM). Participants underwent brachial–ankle pulse wave velocity (baPWV) as well as autonomic function evaluations encompassing the Sudoscan-based modified composite autonomic scoring scale (CASS), baroreflex sensitivity, and heart rate variability in time domains and frequency domains. Linear regression analysis was performed to evaluate the influence of independent variables on baPWV and modified CASS. Participants with higher baPWV values were older, with longer diabetes duration, lower body weight, body mass index, waist circumference, elevated systolic and diastolic blood pressure, and mean arterial blood pressure. They also exhibited a higher prevalence of retinopathy as the underlying disease and reduced estimated glomerular filtration rate. Multiple linear regression analysis revealed that age and BRS were significantly associated with baPWV while diabetes duration, UACR, and BRS were significantly associated with modified CASS. Our study confirms the significant association of BRS with baPWV and modified CASS in T2DM, highlighting its pivotal role in linking microvascular and macrovascular complications. This supports BRS as a surrogate marker for assessing both the severity of arterial stiffness and cardiovascular autonomic neuropathy in T2DM, enabling the early identification of complications.

## 1. Introduction

Diabetes presents a global health challenge, leading to a range of complications including microvascular (nephropathy, retinopathy, and neuropathy) and macrovascular (coronary artery diseases, stroke, and peripheral artery diseases) issues [1,2,3]. Research highlights a notable link between cardiovascular autonomic neuropathy (CAN) and increased mortality risk in diabetes [4]. Our recent study further reveals that the severity of CAN predicts subsequent three-point major adverse cardiovascular events, as evidenced by a 6-year follow-up study [5].

Brachial–ankle pulse wave velocity (baPWV) serves as a non-invasive metric for evaluating arterial stiffness and represents a significant marker of cardiovascular health [6,7]. Elevated baPWV levels correlate with various cardiovascular risks, including hypertension, atherosclerosis, and cardiovascular events, thereby rendering it a pivotal instrument for early detection and risk stratification in clinical contexts [8,9,10].

Baroreflex sensitivity (BRS), assessing the heart rate response to blood pressure fluctuations, reflects the integrity of the autonomic nervous system in modulating cardiovascular functions. The advent of a technique that allows for beat-to-beat blood pressure monitoring in the finger has improved diagnostic capabilities for diabetic patients affected by CAN [4,11]. Our studies further revealed diminished BRS in patients experiencing acute cardiovascular events, obstructive apnea, and cerebral perfusion deficits in Parkinson’s Disease [12,13,14].

Arterial stretch is acknowledged as the primary stimulant of the baroreflex, especially in arterial regions abundant in baroreceptors [15]. Elevated arterial stiffness has the potential to attenuate the activation of baroreceptors, thereby compromising their ability to adequately respond to fluctuations in blood pressure. Previous studies have demonstrated a notable correlation between decreased BRS and heightened baPWV in individuals with congestive heart failure, coronary artery disease, hypertension, and renal impairment [16,17,18,19,20].

The hypothesis posits that baroreflex dysfunction accelerates atherosclerosis by unbalancing the sympathetic and parasympathetic nervous systems [21]. This disequilibrium leads to increased activity in both the sympathetic nervous system and the renin–angiotensin system, while concurrently reducing the activity of the parasympathetic nervous system through inflammatory cascades. As a consequence, this speeds up the development and progression of atherosclerosis [22]. BRS assesses the heart rate’s responsiveness to blood pressure alterations. Increased arterial stiffness may impair baroreceptor activation, compromising their ability to adequately respond to changes in blood pressure. This impairment can lead to baroreflex dysfunction. Consequently, there exists a bidirectional relationship between baroreflex sensitivity and atherosclerosis, wherein each can influence and exacerbate the other, enhancing the feedback loop between them.

The evidence presented elucidates the complex interplay and underscores the fundamental role of BRS in the association between CAN and arterial stiffness. The objective of our study was to determine the role of BRS as a surrogate biomarker for concurrently assessing the severity of arterial stiffness and CAN in individuals with type 2 diabetes mellitus (T2DM).

## 2. Patients and Methods

### 2.1. Study Participants

A prospective case–control study was undertaken at a tertiary healthcare facility and primary referral hospital, aimed at examining the baPWV and conducting autonomic function tests among individuals diagnosed with T2DM. The research encompassed a cohort of 321 individuals diagnosed with type 2 diabetes mellitus (T2DM) who were under observation at the outpatient clinic for a duration exceeding 6 months. Exclusion criteria comprised individuals experiencing moderate-to-severe heart failure (classified under New York Heart Association class III and IV), those presenting with any form of arrhythmia impeding the analysis of heart rate variability (HRV), or individuals with a history of pacemaker implantation for any reason, in addition to those demonstrating significant cognitive impairment. Ultimately, the study enrolled a total of 298 patients diagnosed with T2DM. Authorization for the study was granted by the institutional review board of the hospital (Approval No. 202002095B0). After receiving this authorization, informed consent was secured from all individuals participating in the research.

### 2.2. Baseline Clinical and Laboratory Assessments

Upon initial enrollment, all participants were subjected to an exhaustive evaluation conducted by neurologists with extensive experience. This comprehensive assessment included detailed neurological and physical examinations, encompassing both baseline clinical assessments and laboratory measurements. The gathered data covered a range of variables, such as age at the onset of the disease, sex, height, waist circumference, body mass index (BMI), the duration of the disease, readings of systolic and diastolic blood pressure, cardiometabolic risk factors, and both microvascular and macrovascular complications. Furthermore, for each participant, the urinary albumin-to-creatinine ratio (UACR) [23] and the estimated glomerular filtration rate (eGFR) were determined [24], as has been detailed previously in the literature.

### 2.3. Measurement of baPWV

BaPWV was measured using the BP203RPE-II arteriosclerosis detection device (VP-1000; Omron, Kyoto, Japan) [25]. Participants were asked to avoid caffeinated drinks, tobacco, and alcohol for 30 min before the tests. Measurements were taken in a supine position after at least five minutes of rest, with occlusive cuffs placed on both arms and ankles. Electrocardiograms, phonograms, pulse volume, arterial pressure, and heart rate were recorded simultaneously. BaPWV calculations involved measuring the distance between the brachial and posterior tibial arteries and dividing this value by the time elapsed between these measurements. The resulting figure was then adjusted to account for variations in patient height to ensure accuracy. Averages from both sides were used for analysis. A single experienced operator, blind to participant information, conducted all procedures.

### 2.4. Assessment of Autonomic Function: CASS, BRS, HRV, and ESC Measurements

In order to minimize the influence of circadian rhythm effects, autonomic function testing was conducted between 9:00 am and 12:00 pm. Prior to the test, patients were asked to refrain from consuming any substances that could affect the results. This included medications that could impact autonomic testing. Patients were asked to cease their usage for a period equivalent to five half-lives. This ensured patient safety and well-being.

To assess the severity of autonomic dysfunction, we employed the composite autonomic scoring scale (CASS) developed by Low [26], utilizing its cardiovagal, adrenergic, and sudomotor sub-scores. The evaluation encompassed a test battery including heart rate response to deep breathing (HR_DB), Valsalva ratio (VR), 5 min head-up tilt (HUT) tests, and a quantitative sudomotor axon reflex test (QSART), as outlined by Low. In our institution, we developed a SUDOSCAN-based sudomotor sub-score as a substitute for the QSART [27]. This substitute, along with the cardiovagal and adrenergic sub-scores, enabled the calculation of the modified CASS. Similar to the original CASS score, this modified version also ranges from 0 to 10 points [28]. In addition to CASS, BRS was evaluated using the Valsalva method (BRS_VM). Resting electrocardiographic readings and continuous blood pressure recordings were acquired in the interval between the Valsalva maneuver (VM) and tilt table tests. The baroreflex sensitivity associated with the Valsalva maneuver (BRS_VM) was determined by analyzing the changes in heart rate and blood pressure during the early phase II of the VM, utilizing a least-squares regression analysis.

HRV assessment involved conducting a five-minute resting electrocardiogram (ECG) recording while the patient lay supine with closed eyes. Time domain analysis was utilized to determine the standard deviation of all normal RR intervals (SDNN), whereas frequency domain analysis yielded parameters including high frequency (HF), low frequency (LF), and very low frequency (VLF). Both LF and HF components were computed in absolute power (ms²) as well as in normalized units (n.u.). The LF/HF ratio served as an indicator of sympathovagal balance [27].

### 2.5. Statistical Analysis

Comparative analyses of categorical variables were conducted employing the chi-squared test or Fisher’s exact test as appropriate. Continuous variables were delineated as mean ± standard deviation (SD) or median with interquartile range (IQR), depending on their distribution. The statistical evaluation encompassed three distinct analyses. Initially, the assessment of trends across multiple groups for variables adhering to a normal distribution was undertaken using one-way analysis of variance (ANOVA) with linear polynomial contrasts. Subsequently, a correlation analysis was employed to investigate the associations between baPWV and the modified CASS, alongside variables such as age, the duration of diabetes, body mass index (BMI), waist circumference, peripheral blood assessments for vascular risk factors, and autonomic parameters excluding the modified CASS. The third phase involved executing multiple linear regression analyses to discern the impact of independent variables on baPWV and the modified CASS, separately. Inclusion in the regression model was contingent upon variables demonstrating a significant correlation with baPWV and the modified CASS (*p* < 0.05). For the purposes of data analysis, SPSS Statistics software (version 23, IBM; Redmond, WA, USA) was deployed.

## 3. Results

### 3.1. General Characteristics of Patients with Diabetes

The study encompassed 298 patients diagnosed with T2DM, comprising 141 women (age range: 46–85 years; mean age: 69.1 years) and 157 men (age range: 46–90 years; mean age: 69.2 years). Hypertension (74.8%), hyperlipidemia (52.7%), and stroke (22.5%) emerged as the predominant underlying conditions, with coronary heart disease (7.0%) and peripheral artery diseases (4.0%) following in prevalence. Patient attributes and complication rates at the final assessment are delineated in Table 1 and Table 2, categorized based on ascending quartiles of baPWV. Participants with higher baPWV values were characterized by older age (*p* < 0.001), a longer duration of diabetes (*p* = 0.001), lower body weight, BMI, and waist circumference (*p* < 0.001, *p* < 0.001, and *p* = 0.044, respectively), elevated systolic and diastolic blood pressure, and mean arterial blood pressure (*p* = 0.014, *p* = 0.029, and *p* = 0.013, respectively), a higher prevalence of retinopathy as the underlying disease (*p* = 0.005), and reduced estimated glomerular filtration rate (eGFR) (*p* = 0.028).

### 3.2. The Relationship between baPWV and Autonomic Function

The association between baPWV and autonomic function was investigated in patients with T2DM, categorized based on ascending quartiles of baPWV, as presented in Table 3. Individuals with higher baPWV exhibited lower Valsalva ratio (*p* = 0.03) in modified CASS, and decreased BRS values (*p* < 0.0001). Other parameters did not demonstrate statistical significance.

### 3.3. Effect of Autonomic Function and Other Vascular Risk Factors on baPWV and Modified CASS in Patients with T2DM

The correlation analysis examining the influence of autonomic function and other vascular risk factors on baPWV and modified CASS is presented in Table 4. The statistical outcomes for baPWV (correlation coefficient, P-value) were as follows: age (r = 0.4, *p* < 0.0001), diabetes duration (r = 0.2, *p* < 0.0001), BMI (r = −0.22, *p* < 0.001), systolic blood pressure (SBP) (r = 0.191, *p* < 0.0001), diastolic blood pressure (DBP) (r = 0.102, *p* = 0.042), mean arterial pressure (MAP) (r = 0.12, *p* = 0.03), UACR (r = 0.12, *p* = 0.004), eGFR (r = −0.17, *p* = 0.004), SDNN (r = −0.19, *p* = 0.02), and BRS_VM (r = −0.28, *p* = 0.005).

The statistical findings for modified CASS (correlation coefficient, *p*-value) were as follows: diabetes duration (r = 0.39, *p* < 0.0001), pulse pressure (r = 0.23, *p* = 0.007), UACR (r = 0.3, *p* < 0.0001), eGFR (r = −0.33, *p* < 0.0001), SDNN (r = −0.29, *p* < 0.0001), LF/HF ratio (r = −0.2, *p* = 0.01), and BRS_VM (r = −0.31, *p* < 0.0001).

### 3.4. Clinical Factors Are Significantly Associated with baPWV and Modified CASS in Patients with Diabetes

In the multiple linear regression analysis, only variables significantly correlated with baPWV and modified CASS, respectively, as shown in Table 4, were included. Subsequently, we employed multiple linear regression to identify key determinants underlying baPWV and modified CASS in diabetic patients. The analysis indicated that age and BRS_VM were significantly associated with baPWV, while diabetes duration, UACR, pulse pressure, and BRS_VM were significantly associated with modified CASS (Table 5).

## 4. Discussion

Our study validated the hypothesis that BRS plays a crucial role not only in linking the severity of CAN but also in connecting the severity of arterial stiffness. In addition to cardiovascular reflex tests [29] and CASS systems [26], BRS can function as a readily applicable, non-invasive, and replicable metric for evaluating CAN. Its intrinsic characteristics render it a biomarker of microvascular complications (e.g., CAN), while also serving as a surrogate biomarker for assessing the severity of both microvascular and macrovascular complications.

Numerous studies have employed diverse methodologies to evaluate both cardiovascular autonomic function and arterial stiffness. Shah et al. and Chorepsima et al. validated these findings by demonstrating that diabetic individuals with cardiac autonomic dysfunction, as determined by HRV, displayed elevated pulse wave velocity compared to those without such dysfunction [30,31]. Additionally, Bagherzadeh et al. documented a significant correlation between HRV and arterial stiffness, serving as a measure of atherosclerosis in diabetic patients [32]. Wu et al. illustrated that increased BaPWV was significantly associated with CAN, as evaluated through cardiovascular reflex tests based on Ewing’s protocol, in individuals with T2DM [33]. Furthermore, prior investigations have evidenced a strong correlation between impaired cardiac autonomic function and heightened arterial stiffness in patients diagnosed with T1DM [34,35]. Consistent with earlier research, our findings suggest a significant association between BRS and the severity of arterial stiffness in individuals with T2DM.

The baroreflex mechanism governs both vagal and sympathetic outflows to the heart and blood vessels. A decrease in BRS indicates compromised vagal reflexes, which can result in sustained adrenergic activation [36,37]. BRS holds promise as a comprehensive metric for evaluating cardiovascular autonomic function. In clinical settings, BRS serves as a straightforward, commonly utilized, non-invasive, and reproducible measurement for assessing CAN [38,39]. Previous research has highlighted BRS as an effective diagnostic tool for CAN, characterized by high sensitivity and specificity [40]. BRS analysis could be a valuable method for the early detection of CAN and prove to be a precise screening instrument for the staging and monitoring of CAN in patients with diabetes mellitus; furthermore, reduced BRS at baseline has long-term cardiovascular predictive value in patients with T2DM without structural heart disease [41,42]. As a chronic microvascular complication of diabetes, CAN presents with various clinical manifestations and is associated with an increased risk of mortality [38,39,43]. The findings of this study indicate a significant correlation between elevated baPWV and diminished BRS, further substantiating the relationship between arterial stiffness and CAN. This finding further explains the increased cardiovascular disease risk observed in individuals with T2DM and CAN.

The pathogenesis of microvascular and macrovascular disease in diabetes is complex, involving multiple factors, among which is the compromised capacity of the vascular endothelium to vasodilate, attributed to the inhibition of the nitric oxide (NO) pathway, a prevalent contributing factor [44]. Animal studies indicate that diabetes impairs NO-mediated vasorelaxation, tipping the balance towards vasoconstriction. NO, a key signaling molecule in inflammation, normally acts as an anti-inflammatory agent. Yet, in conditions of excessive production, it becomes a pro-inflammatory mediator, promoting inflammation [45]. This modification can lead to the decreased perfusion of nerve tissue, a conclusion supported by studies on sural nerve biopsy specimens from patients with different severities of neuropathy. These studies uncover progressive structural alterations in nerve microvasculature, such as the thickening of the basement membrane, the degeneration of pericytes, and the hyperplasia of endothelial cells. Arterio-venous shunting also plays a role in diminishing endoneurial perfusion [46]. Moreover, the reduced capillary blood flow to C fibers, encompassing autonomic nerves, results in lowered nerve perfusion and subsequent endoneurial hypoxia [47].

Various cardiovascular risk factors, including hypertension, smoking, obesity, elevated triglyceride levels, and pre-existing cardiovascular disease, are associated with diabetic neuropathy, worsening both microvascular and macrovascular complications [48]. The EURODIAB study found a significant link between CAN development and systolic blood pressure [49]. Excluding autonomic symptoms and function test results from the definition of neuropathy reduced the statistical significance of hypertension’s effect on neuropathy incidence, indicating its robust role as a risk factor. Effective hypertension control, as demonstrated in the United Kingdom Prospective Diabetes Study, not only improved macrovascular outcomes but also decreased microvascular complication incidence [46].

Numerous studies have illustrated that those elevated levels of lipoproteins, particularly low-density lipoprotein (LDL) and very low-density lipoprotein (VLDL), can exacerbate arterial stiffness by promoting endothelial dysfunction and accelerating atherosclerotic changes. This relationship may be mediated through inflammatory pathways that contribute to vascular remodeling and increased arterial rigidity [50,51]. Previous studies have indicated a correlation between dyslipidemia and CAN, and have proposed a potential pathophysiological link between subclinical inflammation, endothelial dysfunction, and cardiac autonomic dysfunction in patients with T2DM [52,53,54]. Conversely, our correlation analysis did not demonstrate a significant association between lipid profiles and baPWV or modified CASS. This discrepancy may be explained by the relatively low levels of lipid profiles observed in the present study, which contrasts with the levels observed in prior studies.

Albuminuria has been demonstrated to exhibit a notable correlation with arterial stiffness and autonomic dysfunction in individuals diagnosed with T2DM [55,56]. It is widely acknowledged that arterial stiffness is influenced by numerous factors, such as advanced age, hypertension, and the duration of diabetes [57,58,59]. Furthermore, prior investigations have established an association between obesity and heightened arterial stiffness [60,61]. Nevertheless, the association between baPWV and BMI or waist circumference exhibits variability. Studies by Huang et al. and Hu et al. have illustrated an inverse correlation between BMI and baPWV among hypertensive individuals, with higher BMI serving as a protective factor against arteriosclerosis, particularly within the age bracket of 35–55 years [62,63]. Tang et al. and Kim et al. have reported a notable inverse association between BMI and baPWV after the adjustment for confounding variables, thus proposing that the obesity paradox could elucidate this finding [64,65]. Our investigation noted a declining pattern in BMI and waist circumference concurrent with an increase in baPWV, aligning with findings in the current body of literature. In addition to considering the obesity paradox, the inverse correlation between baPWV and BMI could stem from BMI not being the most suitable metric for assessing obesity. This suggests the necessity for further investigation to delve into their interrelationship.

## 5. Study Limitations

Our study possessed two primary limitations. Firstly, it adopted a cross-sectional observational design to investigate the potential role of BRS in bridging the severity of CAN and the severity of arterial stiffness. While it provided insight into cardiovascular risk, we did not substantiate cardiovascular events in real-world clinical practice through longitudinal analysis. However, a recent study of ours demonstrated that the severity of CAN serves as a predictive factor associated with subsequent three-point major adverse cardiovascular events, as evidenced by a 6-year follow-up study [5]. Secondly, we did not explore baroreflex dysfunction in terms of inflammation to accelerate atherosclerosis, although a recent study of ours indicated that oxidative stress is implicated in the severity of CAN in individuals with T2DM and prediabetes [66]. Thirdly, our study did not assess plasma acetylcholine and catecholamines nor did it evaluate sympathetic and parasympathetic tones. Furthermore, plasma nitric oxide levels, a significant vasodilator influencing endothelial function, were not measured according to our study design. Incorporating these biomarkers could augment the comprehensiveness of our study, and we intend to address them in future research endeavors. Finally, while arterial stiffness may indeed be influenced by the overall cardiac index of the patients, it is imperative to note that such measurements were not routinely conducted in our study protocol. The incorporation of these biomarkers could significantly enhance the comprehensive nature of our investigation. Consequently, we intend to address this aspect in forthcoming research endeavors.

## 6. Conclusions

Our study affirms the substantial correlation of BRS with baPWV and modified CASS in individuals with T2DM, underscoring its critical role in elucidating the interplay between microvascular and macrovascular complications in this population. This discovery underscores the utility of BRS as a surrogate marker for simultaneously evaluating the severity of arterial stiffness and CAN in individuals with T2DM. Implementing routine BRS assessment among individuals with diabetes not only aids in the early detection of microvascular complications but also facilitates the identification of macrovascular complication risks associated with T2DM.

## Figures and Tables

**Table 1:** Characteristics of patients with type 2 diabetes stratified by ascending quartiles of brachial–ankle pulse wave velocity.

	1st (n = 67)	2nd (n = 74)	3rd (n = 74)	4th (n = 83)	Total (n = 298)	*p*-Value for Trend
**Characteristics**						
Age (year)	64.0 ± 7.6	67.5 ± 8.6	71.6 ± 5.9	73.2 ± 7.2	69.3 ± 8.2	<0.0001 **
Sex (male/female)	34/33	37/37	45/29	41/42	157/141	0.45
Diabetes duration (year)	7.4 ± 5.5	7.9 ± 6.3	9.7 ± 9.3	12.5 ± 9.8	9.5 ± 8.8	0.001 **
Height (cm)	162.5 ± 7.6	161.3 ± 9.0	161.4 ± 7.2	160.2 ± 7.0	161.3 ± 7.7	0.349
Body weight (Kg)	72.8 ± 12.5	72.8 ± 15.1	67.8 ± 11.8	65.1 ± 11.3	69.4 ± 13.1	<0.0001 **
Body mass index (kg/m^2^)	27.9 ± 4.2	27.4 ± 4.9	26.0 ± 3.7	25.4 ± 4.2	26.6 ± 4.4	<0.0001 **
Waist circumstance (cm)	93.1 ± 10.5	96.2 ± 12.6	91.9 ± 9.0	91.6 ± 10.9	93.2 ± 10.9	0.044 *
SBP (mmHg)	132.4 ± 16.0	138.9 ± 19.3	141.1 ± 19.4	141.5 ± 19.6	138.7 ± 19.0	0.014 *
DBP (mmHg)	74.2 ± 10.6	78.7 ± 11.4	79.3 ± 10.8	79.1 ± 13.3	77.9 ± 11.7	0.029 *
MAP (mmHg)	93.6 ± 11.7	98.8 ± 13.3	99.9 ± 12.7	99.9 ± 14.6	98.2 ± 13.4	0.013 *
Pulse pressure (mmHg)	58.2 ± 10.3	60.2 ± 12.2	61.8 ± 13.7	62.4 ± 12.2	60.8 ± 12.3	0.161
BaPWV (cm/s)	1288.1 ± 180.0	1549.5 ± 65.6	1761.0 ± 65.5	2146.6 ± 247.3	1709.6 ± 351.1	<0.0001 **
**Baseline underlying disease**						
Hypertension (%)	52 (77.6%)	54 (73.0%)	54 (73.0%)	63 (75.9%)	223 (74.8%)	0.897
Hyperlipidemia (%)	38 (56.7%)	38 (51.4%)	40 (54.1%)	41 (49.4%)	157 (52.7%)	0.824
Coronary heart disease (%)	4 (6.0%)	5 (6.8%)	6 (8.1%)	6 (7.2%)	21 (7.0%)	0.967
Ischemic stroke (%)	10 (14.9%)	16 (21.6%)	20 (27.0%)	21 (25.3%)	67 (22.5%)	0.287
Peripheral artery disease (%)	3 (4.4%)	2 (2.7%)	2 (2.7%)	5 (6.0%)	12 (4.0%)	0.663
Retinopathy (%)	2 (2.9%)	11 (14.8%)	12 (16.2%)	20 (24.1%)	45 (15.1%)	0.004 **

Data are presented as means ± standard deviations or n (%).* = *p* < 0.05; ** *p* < 0.01. Abbreviations: n, number of cases; SBP, systolic blood pressure; DBP, diastolic blood pressure; MAP, mean arterial pressure, BaPWV, brachial–ankle pulse wave velocity.

**Table 2:** Laboratory test findings of patients with type 2 diabetes stratified by ascending quartiles of brachial–ankle pulse wave velocity.

	1st (n = 67)	2nd (n = 74)	3rd (n = 74)	4th (n = 83)	Total (n = 298)	*p*-Value for Trend
**Laboratory test findings**						
Total cholesterol (mg/dL)	160.2 ± 27.7	160.6 ± 28.9	161.8 ± 29.5	157.0 ± 29.4	159.8 ± 28.8	0.764
Triglyceride (mg/dL)	135.7 ± 73.2	137.4 ± 78.8	143.3 ± 77.9	137.5 ± 80.4	138.5 ± 77.4	0.944
HDL-C (mg/dL)	51.6 ± 16.4	46.8 ± 14.7	48.9 ± 15.5	48.5 ± 14.1	48.9 ± 15.2	0.315
LDL-C (mg/dL)	88.9 ± 39.3	95.3 ± 36.0	86.1 ± 32.3	85.0 ± 31.2	88.7 ± 34.7	0.273
UA (mg/dL)	6.0 ± 1.7	6.2 ± 2.0	6.1 ± 1.4	6.2 ± 1.7	6.1 ± 1.7	0.899
Index HbA1c (%)	7.2 ± 1.0	7.0 ± 0.9	7.2 ± 1.1	7.2 ± 1.0	7.1 ± 1.0	0.363
UACR (mg/g)	9.0 (5.7, 19.2)	10.4 (5.7, 30.4)	11.9 (6.6, 26.5)	19.7 (8.8, 90.0)	11.7 (6.3, 33.0)	0.212
eGFR (mL/min/1.73 m^2^)	79.1 ± 23.1	70.2 ± 27.8	72.3 ± 24.3	65.5 ± 32.5	71.4 ± 27.7	0.028 *

Data are presented as means ± standard deviations or n (%) or median with interquartile range. * = *p* < 0.05. Abbreviations: n, number of cases; HDL-C, high-density lipoprotein cholesterol; LDL-C, low-density lipoprotein cholesterol; HbA1c, glycohemoglobin; eGFR, estimated glomerular filtration rate; UACR, urine albumin–creatinine ratio.

**Table 3:** Autonomic study in patients with type 2 diabetes stratified by ascending quartiles of brachial–ankle pulse wave velocity.

	1st (n = 67)	2nd (n = 74)	3rd (n = 74)	4th (n = 83)	*p*-Value for Trend
**Modified CASS**	1.4 ± 1.2	1.9 ± 1.6	1.8 ± 1.5	2.1 ± 1.5	0.309
Valsalva ratio	1.36 ± 0.20	1.28 ± 0.18	1.27 ± 0.15	1.23 ± 0.11	0.03 *
HRDB	8.2 ± 3.7	7.6 ± 6.1	8.2 ± 6.1	7.0 ± 4.5	0.701
BP change related to standing (mmHg)	−3 (−10, 2.3)	−2 (−12, 3)	−8.5(−17, 0.3)	−4 (−14, 2)	0.57
Sudoscan					
Hands ESC (µS)	53.5 ± 18.6	49.7 ± 20.4	50.7 ± 19.6	48.0 ± 20.5	0.392
Feet ESC (µS)	56.9 ± 16.7	54.3 ± 17.3	53.3 ± 21.0	50.3 ± 18.7	0.183
**Heart rate variability**					
Time domain					
SDNN (ms)	22.3 ± 9.7	19.1 ± 11.7	18.7 ± 12.5	16.0 ± 7.7	0.101
Frequency domain					
LF/HF ratio	0.9 (0.5, 1.9)	1.5 (0.6, 2.9)	1.2 (0.4, 2.5)	0.6 (0.3, 1.2)	0.05
**Baroreflex sensitivity methods**					
BRS_VM (ms/mmHg)	2.6 ± 1.6	2.3 ± 1.3	1.9 ± 0.9	1.7 ± 0.8	<0.0001 **

Data are presented as means ± standard deviations or median (IQR) n (%). * = *p* < 0.05; ** *p* < 0.01. Abbreviations: IQR, interquartile range; n, number of cases; CASS, composite autonomic scoring scale; ESC, electrochemical skin conductance; SDNN, standard deviation of normal RR interval; LF, low frequency; HF, high frequency; n.u., normalized unit; BRS_VM, baroreflex sensitivity was evaluated using the Valsalva method.

**Table 4:** Correlation analysis of baPWV and modified CASS in patients with type 2 diabetes.

Variables	baPWV		Modified CASS	
	r	*p*-Value	r	*p*-Value
Age (year)	0.398	<0.0001 **	0.067	0.442
Diabetes duration (year)	0.204	<0.0001 **	0.386	<0.0001 **
Body mass index (kg/m^2^)	−0.222	<0.0001 **	0.036	0.684
Waist circumstance (cm)	−0.078	0.19	0.133	0.133
SBP (mmHg)	0.191	<0.0001 **	0.147	0.058
DBP (mmHg)	0.102	0.042 *	−0.03	0.69
MAP (mmHg)	0.12	0.03	0.048	0.533
Pulse pressure (mmHg)	0.11	0.07	0.232	0.007 *
Total cholesterol(mg/dL)	−0.06	0.31	0.012	0.89
Triglyceride(mg/dL)	0.009	0.88	0.04	0.68
HDL-C (mg/dL)	−0.080	0.17	−0.08	0.37
LDL-C (mg/dL)	−0.05	0.41	−0.05	0.57
Index HbA1c (%)	0.03	0.59	0.11	0.22
UACR (mg/g)	0.12	0.004 *	0.29	<0.0001 **
eGFR (mL/min/1.73 m^2^)	−0.17	0.004 *	−0.33	<0.0001 **
Modified CASS	0.12	0.11	−−	--
SDNN (ms)	−0.19	0.02 *	−0.29	<0.0001 **
LF/HF ratio	−0.08	0.33	−0.20	0.01 *
BRS_VM (ms/mmHg)	−0.24	0.005*	−0.31	<0.0001 **

r: correlation coefficient. * Indicates that *p*-value < 0.05. ** indicates that *p*-value < 0.0001. Abbreviations: SBP, systolic blood pressure; DBP, diastolic blood pressure; MAP: mean arterial pressure; HDL-C, high-density lipoprotein cholesterol; LDL-C, low-density lipoprotein cholesterol; HbA1c, glycohemoglobin; eGFR, estimated glomerular filtration rate; UACR, urine albumin–creatinine ratio; BRS_VM, baroreflex sensitivity was evaluated using the Valsalva method.

**Table 5:** Effects of the variables on baPWV and modified CASS in patients with type 2 diabetes according to correlation analysis.

	Regression Coefficient	Standard Error	*p*-Value	95% Confidence Interval
Severity of arterial stiffness (baPWV)				
Constant	622.35	203.58	0.003	220.38–1024.68
Age (year)	16.28	2.81	<0.0001	10.73–21.83
BRS_VM (ms/mmHg)	−36.47	15.58	0.021	−67.24–−5.70
Severity of CAN (Modified CASS)				
Constant	0.48	0.89	0.59	−1.28−2.25
Diabetes duration (year)	0.06	0.019	0.002	0.022–0.097
UACR (mg/g)	0.002	0.001	<0.0001	0.001–0.003
Pulse pressure (mmHg)	0.04	0.14	0.013	0.008–0.063
BRS_VM (ms/mmHg)	−0.25	0.10	0.015	−0.44–−0.049

Regression coefficient for each individual variable. Abbreviations: CAN: cardiovascular autonomic neuropathy.

## Data Availability

The data from this study can be acquired from the corresponding author upon reasonable request.
